# Evidence and reality: an observational study on pharmacological outcomes in advanced head and neck tumors

**DOI:** 10.3389/fonc.2025.1598125

**Published:** 2025-07-25

**Authors:** Consuelo Jordán de Luna, Anna Esteve, Olalla Montero Pérez, Ana Clopés Estela, Marc Oliva, Alicia Lozano Borbalas, Ricard Mesía Nin

**Affiliations:** ^1^ Pharmacy Department, Institut Català d’Oncologia-ICO, L’Hospitalet de Llobregat, Barcelona, Spain; ^2^ Department of Clinical Sciences, School of Medicine and Health Sciences, Universitat de Barcelona, Barcelona, Spain; ^3^ Catalan Institute of Oncology, Scientific Knowledge and Research Department, Barcelona, Spain; ^4^ Catalan Institute of Oncology, Scientific Knowledge and Research Department, Pharmacy and Drug Policy Department, Barcelona, Spain; ^5^ Department of Medical Oncology, Catalan Institute of Oncology (ICO), L’Hospitalet de Llobregat, Barcelona, Spain; ^6^ Department of Radiation Oncology, Catalan Institute of Oncology (ICO), L’Hospitalet de Llobregat, Barcelona, Spain; ^7^ Mesía R, Medical Oncology Department, Catalan Institute of Oncology, B-ARGO-group, Institut de Recerca Germans Trías i Pujol (IGTP), Badalona, Spain

**Keywords:** HNSCC, head and neck cancer, real-life data, treatment algorithms, decision-making

## Abstract

The Catalan Institute of Oncology (ICO) is a comprehensive centre for the prevention, diagnosis, and treatment of cancer. At the institute, there is a set of institutional clinical practice guidelines, named ICOPraxis. The field of oncology therapeutics is currently undergoing a transition towards a model of personalised medicine. Institutional clinical guidelines can be more precise in their recommendations by providing a local focus. An evaluation of the utility of clinical guidelines may be conducted through a descriptive analysis of the selection of treatment and the resulting outcome. Retrospective cohort analysis of patients diagnosed with locally-advanced and recurrent/metastatic HNSCC (stage III to IVc according to 7 and 8th edition TNM-AJCC) and treated at the The Head and Neck Cancer Functional Unit of ICO and Bellvitge University Hospital. This study allows us to describe the outcomes of our head and neck tumour patients with real-life data providing information on the possible dilution of the effect when compared to reference clinical trials.

## 1 Introduction

The field of oncology therapeutics is undergoing a transition to a model of personalized medicine. This is occurring in the context of a robust research effort to determine what clinical trials are best for cancer patients. Clinical guidelines are a tool to help clinicians make treatment decisions. There are several well-known clinical guidelines published by scientific societies on a global level. Although it is recognized that other coexisting variables and factors may also influence the final therapeutic approach at the local level, such as the availability of treatment locally or the ability to finance it, institutional clinical guidelines can be more precise in their recommendations by providing a local focus. An evaluation of the utility of clinical guidelines can be conducted through a descriptive analysis of treatment choices and resulting outcomes (1–5).

The Catalan Institute of Oncology (ICO) is a comprehensive center for the prevention, diagnosis, and treatment of cancer. At the Institute, there is a set of institutional clinical practice guidelines called ICOPraxis (6). Multidisciplinary groups - specialists from each of the Cancer Functional Units who are also members of the Multidisciplinary Tumor Boards- develop these guidelines. The latest evidence is reviewed to provide clear and practical recommendations for diagnostic procedures, treatment, and follow-up. Each recommendation is supported by a level of evidence and a grade, according to the methodology established by the European Society for Medical Oncology. These recommendations are incorporated into all multidisciplinary team protocols. In addition, decisions regarding treatment regimens, including drug dosing and the appropriate schedule, are incorporated into the form of treatment algorithms in electronic prescribing software. This software was developed with three objectives: to facilitate knowledge management, to improve process safety, and to achieve results. Over the course of the project’s 17-year history, 17 clinical practice guidelines (updated regularly based on the latest evidence and changes in the standard of care) have been developed for major onco-hematologic clinical conditions. Once published, the guidelines are made available in PDF format and can be accessed free of charge on the ICO website (6). The project is still ongoing and new guides and updates are still being published (7).

For more than two decades, the Head and Neck Squamous Cell Carcinoma (HNSCC) Guidelines have been an integral part of this project. The Head and Neck Cancer Functional Unit of the ICO and Bellvitge University Hospital (HNCFU ICO-HUB) was established in 2004. Since then, physicians have met weekly to discuss and reach consensus on diagnoses and treatments. Each department presents the clinical history of each patient, and after evaluating the case, the most beneficial treatment plan is determined. In our center, the initiation of treatment depends on the case being presented and approved by the multidisciplinary tumor board (8).

In this scenario, it is interesting to analyze the outcomes of patients treated in an institution where cases are discussed in the functional unit committee based on institutional guidelines. The aim of this study is to describe the efficacy and safety outcomes of patients in our center treated according to institutional guidelines.

## 2 Material and methods

### 2.1 Study design:

Retrospective cohort analysis of patients diagnosed with locally advanced and recurrent/metastatic HNSCC (stage III to IVc according to 7th and 8th edition TNM-AJCC) and treated at the HNCFU ICO-HUB. Data were extracted from the HNCFU prospective database established in 2016. Patients were included between January 1, 2016, and February 1, 2023, when their case was evaluated by the tumor board. Briefly, a documentalist collects the data and the patient’s medical history is also recorded under the supervision of a head and neck cancer specialist. All patients enrolled in the study provided informed consent prior to participation. The study was approved by a research ethics committee (PR144/21, University Hospital of Bellvitge).

### 2.2 Patients

Patients were treated according to ICOPraxis recommendations in five different settings (6). Three treatment strategies were selected for locally advanced HNSCC, and two for recurrent and metastatic disease (8). Adult patients who received at least one course of treatment were included. All patients discussed in our the multidisciplinary tumor board were included in the analysis. There are no exclusion criteria. There are no criteria for patient elimination.

The 3 locally-advanced strategies, and the 2 in recurrent/metastatic strategies selected, included these final 5 cohorts:

- Cohort A (TPF-PRE): patients with diagnosed laryngeal/hypopharyngeal squamous cell carcinoma (SCC) cT3-T4aN0/+M0 candidates for total laryngectomy, treated with TPF (docetaxel, cisplatin, 5-fluorouracil 5-FU) induction chemotherapy as an organ-preservation strategy.
- Cohort B (RT-CET): patients with diagnosed HNSCC (oral cavity/oropharynx/larynx or hypopharynx), stage III-IV who have not received any prior treatment and are ineligible for chemotherapy, treated with a combination of radiotherapy and cetuximab.
- Cohort C (TPF-UN): patients with diagnosed HNSCC (oral cavity/oropharynx/larynx or hypopharynx), unresectable, or resectable but with high disease burden-symptomatic disease, treated with TPF induction chemotherapy.
- Cohort D (R/M-EXT): patients with diagnosed recurrent or metastatic HNSCC (regardless of location) platinum-sensitive, treated with EXTREME regimen (platinum, 5-FU, and cetuximab) both first- and second-line for recurrent or metastatic disease. Patients who were included in the second line of treatment have undergone immunotherapy in the first line.
- Cohort E (R/M-ERB): patients with diagnosed recurrent or metastatic HNSCC (regardless of location) who have previously received platinum with a progression-free interval of less than six months, or who had a contraindication to receiving a platinum or 5-fluorouracil, or who had a performance status of greater than one, treated with weekly cetuximab-paclitaxel. Both first- and second-line treatments for recurrent or metastatic disease have been included.

### 2.3 Clinical parameters

Descriptive statistics were provided for all baseline (including Eastern Cooperative Oncology Group [ECOG]), comparative effectiveness, and safety variables, as appropriate.

Organ Preservation Protocol (Laryngeal and Hypopharyngeal Cancer Candidates for Total Laryngectomy)

- No surgery was performed, except in cases where surgery at the cervical level was required at the end of all treatment (salvage cervical lymphadenectomy).
- No excessive secretions leading to microaspiration or recurrent pneumonias.
- Voice function was not impaired.
- Tracheostomy was not performed, and if performed, was closed. There was no nasogastric tube or percutaneous endoscopic gastrostomy. The percentage of organ preservation at each time point was calculated as those patients with organ preservation out of the total number of patients who were on follow-up

Progression-free survival PFS was defined as the time from the date of treatment initiation until the date of progression, the date of cessation of treatment due to toxicity, the date of death, or the day of the last follow-up, whichever occurred first. Overall survival OS was defined as the time from initiation of treatment until the date of death or the day of the last follow-up, whichever occurred first. probability of time-to-event-free was estimated using the Kaplan–Meier method. The median overall survival and median progression-free survival, along with their 95% confidence intervals (CIs), were reported. Patients who did not experience documented progression or death during the study period were censored on the last date of tumour evaluation.

Safety was evaluated in all cohorts using the National Cancer Institute Common Toxicity Criteria (NCI CTCAEIV, version current at the time of the study). Safety data included a description of adverse events classified as Grade 3-5 (Grade 1 and 2 adverse events are not recorded in the medical record). The percentage toxicity Grade 3 or higher was calculated as number of the total in each cohort.

### 2.3 Variables

Information was collected on the general characteristics of the patient population diagnosed and treated for HNSCC. These include age, sex, smoking and alcohol consumption habits, functional stage, and TNM classification at diagnosis according to the 7th and 8th editions of TNM-AJCC.

Response was assessed using standardized radiographic criteria.

The effectiveness endpoints analysed in each cohort were selected in order to match those measured in the original trial of each treatment regimen (9).

1. Cohort TPF-PRE: larynx preservation among surviving patients at 3 months, 3 and 5 years, progression-free survival (PFS), and overall survival (OS) (10).
2. Cohort RT-CET: locoregional control, PFS, OS (11, 12).
3. Cohort TPF-UN: PFS, OS (13).
4. Cohort R/M-EXT: OS (14).
5. Cohort R/M-ERB: PFS, OS (15).

The total number of patients who experienced an adverse event in each cohort was also reported.

### 2.4 Statistical analysis

Descriptive statistics were provided for all baseline, comparative effectiveness, and safety variables as appropriate. The percentage of organ preservation at each time point was calculated as those patients with organ preservation out of the total number of patients who were on follow-up. The percentage toxicity CTCAE Grade 3 or higher were calculated as number of the total in each cohort.

PFS was defined as the time from the date of treatment initiation until the date of progression, the date of cessation of treatment due to toxicity, the date of death, or the day of the last follow-up, whichever occurred first. OS is defined as the time from initiation of treatment until the date of death or the day of the last follow-up, whichever occurred first The probability of being event-free over time was estimated using the Kaplan–Meier method. The median OS and median PFS, along with their 95% confidence intervals (CIs), were reported. Patients who did not experience documented progression or death during the study period were censored on the last date of tumor evaluation.

The percentages of adverse events were calculated as the total number of patients who experienced the event, relative to the total number of patients in the corresponding cohort.Adverse events were calculated as a percentage of the total number of patients in each cohort.

All statistical analyses were conducted using R software (version 4.1.2).

## 3 Results

### 3.1 Patient disposition

**Figure 1:**
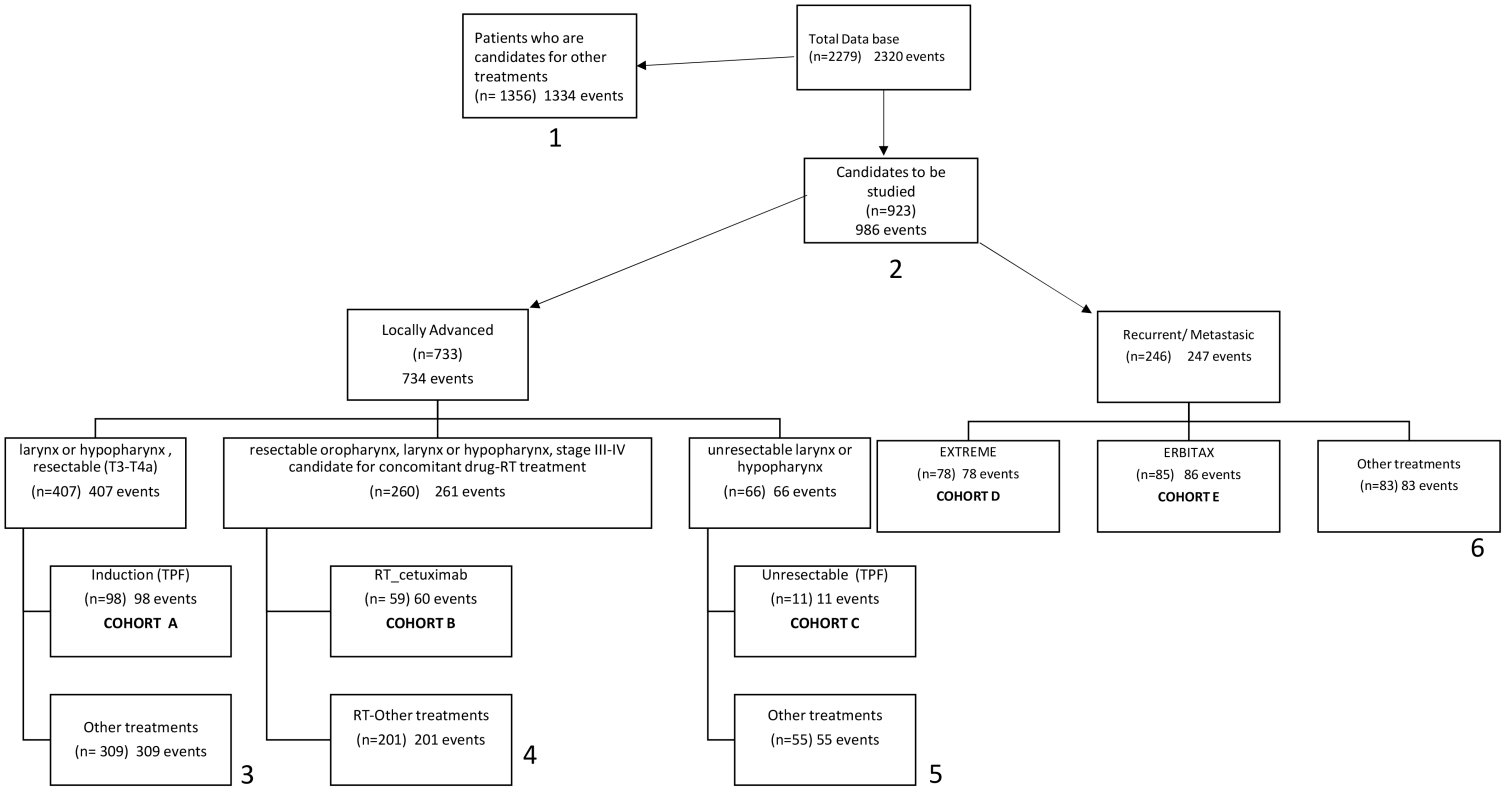
Flow-chart. (1) Other treatments: surgery with or without complementary TT, clinical monitoring, the patient does not want to be treated, Palliative surgical intervention, palliative radiotherapy, non-active treatment. (2) Patients from the candidate group to be studied in locally advanced tumours to the candidate group to be studied in recurrent or metastatic treatment. Cross (n= 56) (64 events). (3) Clinical trial (n= 15), cetuximab-RT (n=18), surgery with or without complementary TT (n=183), the patient does not want to be treated (n= 7), concomitant Ch-RT (n=67), exclusive RT (n=15), treatment at another departamento (n=1). (4) Clinical trial (n= 2), CBDCA (n= 2), CDDP (n= 197). (5) surgery with or without, complementary TT (n=17), clinical trial (n=1), concomitant TT-RT (n=31), exclusive RT (n=6). (6) Nivolumab (n= 20), taxol/weekly (n=4), PF (n=4), clinical trial (n=57). Rt, Radiotherapy; TT, Treatment; Ch, Chemotherapy.

From a database containing 2,279 patients, a total of 312 patients from the HNCU ICO-HUB met the inclusion criteria in 5 treatment cohorts (A: 98, B: 59, C: 11, D: 78, E: 85). The discrepancy between the number of events and the number of patients was attributable to two cases where the same treatment was received for two metachronous head and neck cancers (HNCs) and to the remaining 19 cases where treatments were received for locally advanced disease as well as recurrent/metastatic disease. **Figure 1** illustrates the flowchart.

### 3.2 Demographic and baseline clinical characteristics

The median age of the population under study is 63.3 years. The majority of patients in all cohorts were male (82.3-91.8%). Up to 81.80-95.90% were active or former smokers and a (69.60-80.70%) had a history of alcohol consumption. Eastern Cooperative Oncology Group (ECOG) performance status was mainly of 1 (45.5-71,4%), 0 (8,2 -22.8%) or 2 (1,0-29.4%). (see **Table 1**).

**Table 1:** Baseline characteristics of the patients.

Variable Cohort	TPF-PRE N=98	RT-CET N=60	TPF-UN N=11	R/M-EXT N=79	R/M-ERB N=85
**Age (years),** Mean (SD)	61.0 (6.8)	67.9 (12.8)	56.9 (9.2)	58.3 (9.5)	68.1 (8.7)
Sex, n (%):					
Women	8 (8.2%)	9 (15.0%)	1 (9.1%)	14 (17.7%)	13 (15.3%)
Men	90 (91.8%)	51 (85.0%)	10 (90.9%)	65 (82.3%)	72 (84.7%)
Tobacco, n (%):					
Former smoker (> 1year)	27 (27.6%)	25 (41.7%)	3 (27.3%)	34 (43.0%)	35 (41.2%)
Smoker (< 10 packet-year)	12 (12.2%)	4 (6.7%)	–	7 (8.9%)	9 (10.6%)
Smoker (>10 packet-year)	55 (56.1%)	27 (45.0%)	6 (54.5%)	27 (34.2%)	31 (36.5%)
Non-smoker	3 (3.1%)	3 (5.0%)	1 (9.1%)	11 (13.9%)	8 (9.4%)
‘Missing’	1 (1.0%)	1 (1.7%)	1 (9.1%)	–	2 (2.4%)
Enolism, n (%):					
Alcohol	2 (2.0%)	–	–	1 (1.3%)	–
Alcohol/Mild (<Unit)	17 (17.3%)	6 (10.0%)		9 (11.4%)	9 (10.6%)
Alcohol/Moderate (Unit-Unit)	22 (22.4%)	12 (20.0%)	3 (27.3%)	20 (25.3%)	17 (20.0%)
Alcohol/Severe (>Unit)	19 (19.4%)	10 (16.7%)		15 (19.0%)	20 (23.5%)
Former alcohol (>1 any)/Mild	–	2 (3.3%)	1 (9.1%)	1 (1.3%)	1 (1.2%)
Former alcohol (>1 any)/Moderate	6 (6.1%)	2 (3.3%)	1 (9.1%)	2 (2.5%)	5 (5.9%)
Former alcohol (>1 any)/Severe	13 (13.3%)	10 (16.7%)	2 (18.2%)	7 (8.9%)	10 (11.8%)
No Alcohol	12 (12.2%)	12 (20.0%)	2 (18.2%)	21 (26.6%)	14 (16.5%)
‘Missing’	7 (7.1%)	6 (10.0%)	1 (9.1%)	3 (3.8%)	9 (10.6%)
Performance Status^1^ at diagnosis, n (%):					
0	19 (19.4%)	7 (11.7%)	2 (18.2%)	18 (22.8%)	7 (8.2%)
1	70 (71.4%)	39 (65.0%)	5 (45.5%)	55 (69.6%)	49 (57.6%)
2	1 (1.0%)	14 (23.3%)		3 (3.8%)	25 (29.4%)
3	–	–		1 (1.3%)	2 (2.4%)
‘Missing’	8 (8.2%)	–	4 (36.4%)	2 (2.5%)	2 (2.4%)
Head and Neck tumour main localisation, n (%):					
Hypopharynx	17 (17.3%)	7 (11.7%)	4 (36.4%)	9 (11.4%)	13 (15.3%)
Larynx	81 (82.7%)	17 (28.4%)	7 (63.6%)	19 (24.1%)	18 (21.2%)
Oral cavity **^2^**	–	1 (1.7%)	–	21 (29,2%)	23 (23.6%)
Oropharynx	–	32 (53.3%)	–	21 (26.7%)	24 (28.4%)
Other localizations**^3^**	–	3 (5%)	–	7 (8,9%)	10 (11.9%)
Anatomic stage at diagnostic/Prognostic groups, n (%):					
I	–	–	–	2 (2.5%)^4^	4 (4.7%)^3^
II	–	–	–	5 (6.3%)^4^	4 (4.7%)^3^
III	47 (48.0%)	19 (31.7%)	3 (27.3%)	10 (12.7%)^4^	9 (10.6%)^3^
IVA	37 (37.8%)	35 (58.3%)	7 (63.6%)	36 (45.6%)	34 (40.0%)
IVB	14 (14.3%)	3 (5.0%)	1 (9.1%)	1 (1.3%)	8 (9.4%)
IVC				18 (22.8%)	13 (15.3%)
‘Missing’	–	3 (5.0%)	–	7 (8.9%)	13 (15.3%)

1 ECOG, Eastern Cooperative Oncology Group; 2 Oral cavity, Oral cavity, Mandible, Upper jaw; 3 Nasal carcinoma, Lips, Sinus paranasal, Cervical, Cervical esophagus, Skin, Major salivary glands/Submandibular glands. 4 This is staging at diagnosis in patients who have been treated for recurrent disease at disease progression.

### 3.3 Effectiveness by cohort

**Figure 2:**
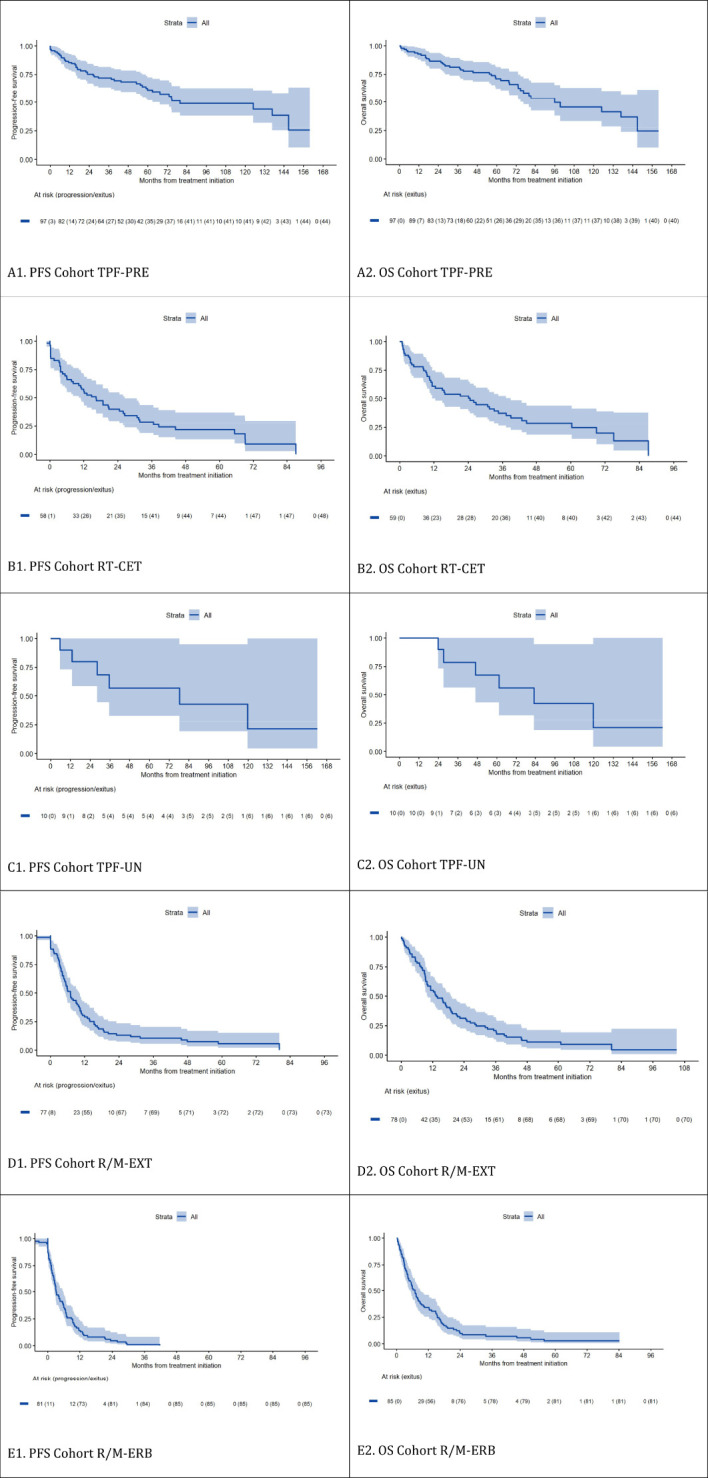
Progression-free Survival and Overall Survival According to the Cohort Group (Kaplan–Meier estimation). shows each cohort. First, progression-free survival and second, overall survival. Thus, **(A1)** corresponds to the PFS of the TPF-PRE cohort and **(A2)** to the OS of that cohort, **(B1)** corresponds to the PFS of the RT-CET cohort and **(B2)** to the OS of that cohort, **(C1)** corresponds to the PFS of the TPF-UN cohort and **(C2)** to the OS of that cohort, **(D1)** corresponds to the PFS of the R/M-EXT cohort and **(D2)** to the OS of that cohort, and finally, **(E1)** corresponds to the PFS of the R/M-ERB cohort and **(E2)** to the OS of that cohort.

The efficacy results were compared with the pivotal studies that support the use of each regimen and are summarized in **Table 2**, **Figure 2**.

**Table 2:** Benchmark studies in the five cohorts studied in head and neck tumour treatment compared to the results of our cohorts.

Reference	Study design	Treatments (n)	Result
Cohort TPF-PRE			
Pointreau Y J Natl Cancer Inst. 2009 (10)	Phase III, randomised. Patient: non-metastatic E III-IV squamous cancer of the larynx or hypopharynx, previously untreated.	-Exptal.: Induction Ch with TPF c/21 d x 3 - Ctrl.: Induction Ch with PF c/21 d x 3 (Total n = 213)	Exptal. (n = 110) *vs.* Ctrl. (n = 103) __________________ Larynx preservation (3 y): 70.3% *vs.* 57.5%; p = 0.03 __________________ OS (3 y): 60% *vs*. 60%; p = 0.57 __________________ Febrile neutropenia: 10.9% vs. 5.8%. Thrombopenia: 1.8% *vs*. 7.8%. Stomatitis: 4.6% *vs*. 7.8%.
Cohort A	Adult patients diagnosed with locally advanced, resectable (T3-T4a), previously untreated laryngeal or hypopharyngeal tumour, candidates for TPF treatment. (n= 98)		Larynx preservation (3 months) 81.1% (n= 95) (3 y) 82.1% (n=67) (5 y) 90.2% (n= 41) __________________ PFS (median) 79.9 months; 95% IC [62.9-NA] PFS (5 y) 61%; 95% CI [52-72] PFS (10 y) 49%; 95% CI [38-63]. __________________ OS (median) 95.9months; 95% CI [76.6-NA]. OS (5 y) 71%; 95% CI [62-81] OS (10 y) 46%; 95% CI [33-63]
Cohort RT-CET			
Bonner et al. N Engl J Med 2006, 2010 (11, 12)	Phase III, randomised. - Patient: carcinoma squamous of oropharynx, hypopharynx or stage larynx III-IV no metastatic.	- Exptal.: RT radical (7–8 wk, high doses) concomitant with cetuximab. - Ctrl: RT radical (7–8 wk, high doses) (Total n = 424)	Exptal. (n= 211) *vs*. Crtl. (n= 213) __________________ Median PFS (m)(2006): 17.1 vs. 12.4; HR = 0.70 (0.54-0.90); p = 0.006 PFS (2 y): 46% *vs*. 37%; p= 0.04 __________________ Median OS (m) (2010): 49.0 vs. 29.3; HR = 0.73 (0.56-0.95); p = 0.018 OS (5 y) 45.6% *vs*. 36.4
Cohort B	Adult patients with a diagnosis of locally advanced resectable oropharyngeal, laryngeal or hypopharyngeal tumour, stage III-IV, previously untreated, candidates for treatment with radiotherapy plus cetuximab. (n= 60)		PFS (median) 16.3 months; 95% IC [10.2-26.4] PFS (3 y) 28%; 95% CI [19–43] PFS (5 y) 22%; 95% CI [13-37]. __________________ OS (median) 24.6; months 95% CI [11.4-38.9]. OS (2 y) 52%; 95% CI [41-67]
Cohort TPF-UN			
Vermorken JB N Engl J Med. 2007 (13)	Phase III, randomised, open. - Patient: squamous cell carcinoma, locally advanced in stages III-IV no metastatic, not treaty previously.	-Exptal.: TPF c/21 d x 4. - Ctrl: PF c/21 d x 4. Both arms, if there is no progression: RT at 4–7 wks post-CT. (Total n=358)	Exptal. (n=177) *vs*. Ctrl. (n=181) __________________ Median PFS (m) (OP): 11 vs. 8.2; p = 0.007 HR = 0.72 (0.57-0.91) __________________ Median OS (m): 18.8 *vs*. 14.5; RH = 0.73 (0.56-0.94); p = 0.02
Cohort C	Adult patients diagnosed with locally advanced, unresectable, larger volume (N3, N2c, massive N2b, T4b) or very symptomatic and fast growing (undifferentiated grade III, fusocellular subtype) laryngeal or hypopharyngeal tumour. Previously untreated, candidates for treatment with TPF (docetaxel, cisplatin, 5-fluorouracil). (n= 11)		PFS (median) 78.4months; 95% IC [28.3-NA] PFS (3 y) 57%; 95% CI [33-100] PFS (5 y) 57%; 95% CI [33-100] PFS (10 y) 21%; 95% CI [4-100] __________________ OS (median); 83 months 95% CI [46.8-NA]. OS (3 y) 80%; 95% CI [59-100] OS (5 y) 57%; 95% CI [33-100] OS (10 y) 21%; 95% CI [4-100]
Cohort R/M-EXT			
Vermorken et al. N Ingl J Med 2008 EXTREME (14)	- Phase III, randomised, open. - Patient: squamous carc. recurrent or metastatic, not previously discussed.	- Exptal.al: FP (1/5-FU Platinum) IC 4 d c/21 of max. x 6) + cetuximab sem. - Ctrl.: PF (1/5-FU IC platinum) 4 d c/21 of max. x 6) (Total n= 442)	Exptal. (n = 222) *vs*. Ctrl (n = 222): __________________ Median PFS (m): 5.6 *vs*. 3.3 HR = 0.54 (0.43-0.67); p ≤0.001 __________________ Median OS (m): 10,1 *vs*. 7,4 HR = 0.80 (0.64-0.99); p = 0.04 Grade 3–4 toxicity: 82% *vs*. 76%; NS
Cohort D	Adult patients with a diagnosis of recurrent or metastatic head and neck tumour (regardless of location), have not received platinum as a primary treatment or who have previously received platinum with a progression-free interval of more than 6 months treated with platinum + 5-FU + cetuximab. Both first- and second-line treatments in recurrent or metastatic disease have been included. (n=79)		PFS (median) 7 months; 95% IC [5.4-10.1] PFS (6 months) 53%; 95% CI [43–65] PFS (1 y) 29%; 95% CI [21–41] PFS (2 y) 13%; 95% CI [7-24] __________________ OS (median); 13.4 months 95% CI [10.1-19.1]. OS (6 months) 79%; 95% CI [71-89] OS (1 y) 55%; 95% CI [45-67] OS (2 y) 31%; 95% CI [23-44]
Cohort R/M-ERB			
Hitt R Ann Oncol. 2012 (15)	Phase II, open, single group	Cetuximab 250 mg/m2 weekly (with D load 400 mg/m2) -paclitaxel 80 mg/m2 weekly (Total n= 46)	OR= 54% (95% CI 39% -69%), CR= 22% (95% CI 11% -36%) PR= 33%. (95% CI 20% - 48%) __________________ PFS= 4.2 months 95% CI [2.9-5.5] OS = 8.1 months 95% CI [6.6-9.6]
Cohort E	Adult patients diagnosed with recurrent or metastatic head and neck tumour (regardless of location), PS performance status 0-2, who have previously received platinum with a progression-free interval of less than 6 months or with contraindication to receiving a platinum (cisplatin or carboplatin) or 5-fluorouracil. (n=85)		PFS (median) 3.2 months; 95% IC [2.5-5.8] PFS (6 months) 35%; 95% CI [26-47)] PFS (1 y) 14%; 95% CI [8-24] PFS (2 y) 5%; 95% CI [2-12] __________________ OS (median) 6.9 months; 95% CI [5.2-8.9]. OS (6 months) 52%; 95% CI [42-64] OS (1 y) 34%; 95% CI [25-46] OS (2 y) 10%; 95% CI [5-19]

Exptal, Experimental; Ctrl, Control; Exptal., Experimental; Ch, Chemotherapy; RT, radiotherapy; TPF, docetaxel/cisplatin/5-FU; PF, cisplatin/5-FU; CDDP, cisplatin; CBDP, carboplatin; CBDP, carboplatin; y, years; m, months; OS, Overall survival; OR, Objective Response; CR, Complete Response; PR, Partial response; FS, Free survival; OR, Overall response

In Cohort A (TPF-PRE), laryngeal preservation was achieved in 82.1% of surviving patients at 3 years and 90.2% at 5 years. Survival outcomes were favorable, with a 10-year OS of 46% and a median PFS close to 80 months. These results compare positively with those reported in the trial by Pointreau et al.

In Cohort B (RT-CET), which includes patients ineligible for cisplatin, the 2-year OS was 52%, and median PFS was 16.3 months. These findings are in line with the outcomes observed in the Bonner et al. trial, though with slightly older patients in our cohort.

In Cohort C (TPF-UN), long-term follow-up showed a 10-year OS and PFS of 21%. While the sample size was small (n = 11), these data are consistent with those reported by Vermorken et al. for similar regimens in unresectable disease.

Cohort D (R/M-EXT), treated with platinum, 5-FU, and cetuximab, showed a median OS of 13.4 months and a 2-year OS rate of 31%, which are comparable to or slightly superior to those in the EXTREME trial.

In Cohort E (R/M-ERB), composed of less fit patients treated with weekly cetuximab and paclitaxel, the median OS was 6.9 months, with a 2-year survival of 10%. This aligns with outcomes from the Hitt et al. study and real-world data, though slightly less favorable OS was observed.

Detailed numerical comparisons with the benchmark studies are presented in **Table 2**.

### 3.4 Safety

Only those adverse events (AEs) graded as grade CTCAE ≥ 3 were collected in the study. The number and percentage of patients who experienced grade CTCAE ≥ 3 treatment-related adverse effects (toxicities) were as follows. (A) 71 (72.4%), (B) 45 (75.0%), (C) 9 (81.8%), (D) 44 (55.7%) and (E) 44 (55.7%). The rest of the toxicities recorded are listed in **Table 3** of this article. The most prevalent grade ≥ 3 toxicities were neutropenia and mucositis. During the aforementioned period, no instances of toxic death have been documented.

**Table 3:** Grade 3 or 4 adverse events in the safety population.

Cohort										
Adverse event	TPF-PRE		RT-CET		TPF-UN		R/M-EXT		R/M-ERB	
CTCAE Grade	Grade 3 &4	Grade 4	Grade 3 &4	Grade 4	Grade 3 &4	Grade 4	Grade 3 &4	Grade 4	Grade 3 &4	Grade 4
	N %	N %	N %	N %	N %	N %	N %	N %	N %	N %
Any event No Missing	71 (72.4%)		45 (75.0%)		9 (81.8%)		43 (50.6%)		44 (55.7%)	
	26 (26.5%)		14 (23.3%)		1 (9.1%)		39 (45.9%)		29 (36.7%)	
	1 (1.0%)		1 (1.7%)		1 (9.1%)		3 (3.5%)		6 (7.6%)	
Non-hematological toxicities										
Mucositis	37 37.8	1 1.0	36 60.0	2 3.3	4 36.4	1 9.1	13 16.5	–	7 8.2	1 1.2
Febrile neutropenia	12 12.2	11 11.2	–	–	2 18.2	2 18.2	4 5.1	1 1.3	2 2.4	2 2.4
Anorexia	1 1.0	0 0	–	–	–	–	3 3.8	–	1 1.2	0 0
Dysphagia	1 1.0	–	2 3.3	–	–	–	1 1.3	–	–	–
Gastrointestinal toxicity	9 9.2	0 0	0 0	0 0.0	1 9.1	–	10 12.7	–	–	–
Skin toxicity^1^	16 16,3	0 0	19 31,7	0 0	4 36,4	1 9.1	12 15.1	0 0	11 13	0 0
Neuropathy	–	–	–	–	–	–	1 1.3	0 0.0	–	–
ungeal disorders	–	–	–	–	–	–	4 5	0 0.0	3 3.5	0 0.0
Chelitis	4 4.1	0 0.0	5 8.3	0 0	–	–	2 2.5	0 0.0	1 1.2	0 0
Odynophagia	19 19.4	0 0.0	13 21.7	0 0	2 18.2	0 0	2 2.5	0 0.0	1 1.2	0 0
Alterations in laboratory results^2^	2 2.0	0 0	4 6,7	0 0	–	–	6 7.7	0 0.0	3 3.6	1 1.2
Infection	–	–	–	–	–	–	2 2.5	2 2.5	2 2.4	0 0
Shock of unfilial aetiology	–	–	–	–	–	–	–	–	1 1.2	0 0
Epithelitis	–	–	–	–	1 9.1	0 0	–	–	–	–
Dysgeusia	3 3.1	0 0	3 5.0	0 0.0	–	–	–	–	–	–
Xerostomia	2 2.0	0 0	1 1.7	0 0	–	–	–	–	–	–
Anaphylaxis due to cetuximab	–	–	1 1.7	1 1.7	–	–	–	–	–	–
Acute renal failure	–	–	1 1.7	0 0	–	–	–	–	–	–
Bilateral hearing loss	–	–	1 1.7	0 0	–	–	–	–	–	–
Haematological toxicities										
Asthenia	5 5.1	1 1.0	5 8.3	0 0	–	–	13 16.5	0 0.0	14 16.5	0 0.0
Anemia	7 7.1	0 0.0	–	–	1 9.1	0 0	5 6.3	0 0.0	6 7.1	1 1.2
Thrombocytopenia	1 1.0	0 0	–	–	1 9.1	0 0	5 6.3	1 1.3	–	–
Haematological toxicity	1 1.0	1 1.0	–	–	–	–	–	–	–	–

The most relevant toxicity or toxicity greater than 5% is represented.1 Skin toxicity: Folliculitis, Radiodermatitis, Rash, Palmar-plantar Erythrodysesthesia, Stretch marks on hands, Dermatitis. 2 Alterations in laboratory results: GGT elevation, Hypophosphatemia, Hyponatraemia, Hyperamylasaemia, Lipase elevation, elevated aminotransferases, Hypomagnesaemia, ALT elevation.

## 4 Discussion

In this article, we demonstrate that the results obtained in our population are similar to those of patients included in different treatment regimens for HNSCC. We believe that this result is closely related to the fact that our institution has a treatment algorithm that serves as a guide for selecting the patient-treatment combination, as explained in the introduction.

With regard to laryngeal preservation outcomes (cohort TPF-PRE), the optimal observed PFS, OS, and toxicity results may be attributed to the fact that the multidisciplinary team, comprising professionals who have been in post since 1994, has been working in a monographic centre. As a reference centre, it handles a relatively high number of patients treated with this scheme and has been perfecting its management with fluid therapy, support medication, and controls in accordance with the established protocol. In the case of patients treated with radiotherapy plus cetuximab (Cohort RT-CET), it is noteworthy that the patients included in this cohort received this treatment as an alternative to the standard radiotherapy plus cisplatin. Our institutional guidelines prioritize concomitant treatment with cisplatin over concomitant treatment with cetuximab. Consequently, the fitter population, as they were originally treated in the Bonner trial (11), has been treated with cisplatin-RT. In our population, compared to that treated in the Bonner study, patients were 10 years older. The relatively small size of Cohort TPF-UN (n = 11) precludes any meaningful analysis of the results at this stage. It is therefore necessary to recruit a larger number of patients in order to obtain a more robust dataset. This is due to the fact that, from the year 2000 onwards, there has been a tendency to utilise induction therapy less frequently in patients with unresectable tumours (in favour of concomitant chemoradiotherapy) (14). Consequently, we only employ this approach when the patient is experiencing significant symptoms, or when there is a risk to the patient’s life if we delay the commencement of radiotherapy. In cohort C, treatment is administered to patients with large tumour volumes (potentially aggressive disease) and non-HPV-related tumours, so it is less common to find oropharyngeal tumours, and this cohort has very few patients, hence the lower incidence of oropharyngeal tumours (16).

In Cohort R/M-EXT (EXTREME regimen), both PFS and OS are comparable to, but slightly superior to, those observed in the landmark study by Vermoken et al. (2008). It is crucial to highlight that our population encompasses patients who have been treated for both first- and second-line metastatic disease, because this regimen moved to the second line with the introduction of immunotherapy in first line. It is also noteworthy that the proportion of patients with oral cavity tumours in our cohort (29.2%) is higher than that observed in the EXTREME arm of the Vermoken et al. study (21%). The results of the subgroup analysis indicate a significantly positive difference in favour of the experimental treatment compared to the standard treatment. This discrepancy is not observed in the other locations under analysis (14).

Ultimately, in unfit patients treated with weekly paclitaxel and cetuximab (Cohort R/M-ERB), the PFS of our cohort is comparable to that observed in the Hitt 2012 study and the real-world evidence analysis published by Rubió et al., with a median of 4.5 months (95% CI: 3.9–5). Conversely, the median OS is less favourable than that observed in the Hitt and Rubió studies. It is notable that a considerable proportion of patients within this cohort had participated in clinical trials, representing approximately 25% of the total group. Consequently, patients who were not eligible for clinical trials, and who might be in a more advanced stage of disease and/or had additional comorbidities, had been excluded from the analysis. This may contribute to the observed trend of slightly inferior OS. It is also important to note that patients who are eligible for this treatment option have palliative care as an alternative. Distinguishing which patients will benefit from treatment, and which will not, is a significant challenge. This cohort will be subject to further review in the future (15, 17).

With regard to toxicity, as this is a retrospective observational study, the collection of toxic events is limited as the information is already collected and confounding factors may not have been taken into account. t should be clearly (especially, specifically) noted that no grade 1 or 2 events were collected. This is an inherent limitation of an observational study, as adverse events of grades 1 and 2 are less frequently reported in such studies than in clinical trials. The most frequently reported adverse events were mucositis (in our series, grade 3, 35.7% and grade 4, 1%, with post-consolidation TREMPLIN data indicating 43% grade 3 and 2% grade 4), odynophagia (only grade 3, with no reports identified in GORTEC or TREMPLIN) and neutropenia (15.3% in our cohort and 31.5% grade 4 or higher in the TPF branch of GORTEC) (10, 18).

A review of the literature shows that clinical guidelines published at the level of scientific societies or national bodies do not include outcome analyses. The contribution of our study is that we have obtained real-life data for head and neck tumour treatments, which have been achieved by an experienced, multidisciplinary team with consistent decision-making and adherence to agreed treatment algorithms. This allows for a comparison with the expected results based on supporting clinical trials.

The evidence base for the benefits and risks of treatments in the health sciences is primarily derived from randomised clinical trials (RCTs). The evidence base for newest treatments is incomplete at the time of approval by regulatory agencies, and in many cases, insufficient for the formulation of well-informed decisions. The utilization of real-life data studies serves to complement the information obtained in clinical trials, or alternatively, to inform decision-making in scenarios where data are lacking. Another valuable application of real-life data studies is the conduct of rational drug use studies. As stated by the World Health Organization (WHO), effective research in drug utilization necessitates the involvement of multiple disciplines, including clinicians, clinical pharmacologists, pharmacists, and epidemiologists. In the absence of the support of prescribers, it is unlikely that this research endeavour will succeed in its objective of facilitating the rational use of drugs (19–21).

The Catalan Institute of Oncology Head and Neck Cancer Unit, has worked as a multidisciplinary team for several years, developing its own clinical guidelines, including the first guidelines for head and neck tumours in 2011. It is considered a favourable context for studying outcomes in patients receiving treatments in routine clinical practice. However, the main limitation of the study is that it is not a controlled trial, as patients were not randomly assigned to follow or not follow the evidence-based clinical guidelines, making a clinical trial illogical in this case (22).

To the best of our knowledge, there are oncology outcome analyses and publications on the experience of decision-making in multidisciplinary teams. However, we have not found any outcome analyses in patients treated by multidisciplinary teams with clinical guidelines published in open access by the teams themselves (5, 23–26).

## 5 Conclusions

This study allows us to describe the outcomes of our head and neck tumor patients with real-life data, providing information on the possible dilution of the effect when compared to reference clinical trials. It is also interesting to have one’s own data history. Although clinical trials remain the gold standard for decision-making, obtaining real-life data helps us in scenarios where clinical trials are not available, or when refining complex decisions in scenarios of uncertainty or when assigning priorities in the design of algorithms. Future studies of real-life data are needed to help knowledge management in an increasingly complex environment.

## Data Availability

The raw data supporting the conclusions of this article will be made available by the authors, without undue reservation.
