# Correction: Evidence and reality: an observational study on pharmacological outcomes in advanced head and neck tumors

**DOI:** 10.3389/fonc.2025.1677769

**Published:** 2025-08-21

**Authors:** Consuelo Jordán de Luna, Anna Esteve, Olalla Montero Pérez, Ana Clopés Estela, Marc Oliva, Alicia Lozano Borbalas, Ricard Mesía Nin

**Affiliations:** ^1^ Pharmacy Department, Institut Català d’Oncologia-ICO, L’Hospitalet de Llobregat, Barcelona, Spain; ^2^ Department of Clinical Sciences, School of Medicine and Health Sciences, Universitat de Barcelona, Barcelona, Spain; ^3^ Catalan Institute of Oncology, Scientific Knowledge and Research Department, Barcelona, Spain; ^4^ Catalan Institute of Oncology, Scientific Knowledge and Research Department, Pharmacy and Drug Policy Department, Barcelona, Spain; ^5^ Department of Medical Oncology, Catalan Institute of Oncology (ICO), L’Hospitalet de Llobregat, Barcelona, Spain; ^6^ Department of Radiation Oncology, Catalan Institute of Oncology (ICO), L’Hospitalet de Llobregat, Barcelona, Spain; ^7^ Mesía R, Medical Oncology Department, Catalan Institute of Oncology, B-ARGO-group, Institut de Recerca Germans Trías i Pujol (IGTP), Badalona, Spain

**Keywords:** HNSCC, head and neck cancer, real-life data, treatment algorithms, decision-making

In the published article, an author name was incorrectly written as “Riard Mesía Nin”. The correct spelling is “Ricard Mesía Nin”.

In the published article, an author name was incorrectly written as “Anna Esteve Gomez”. The correct spelling is “Anna Esteve”.

The authors apologize for this error and state that this does not change the scientific conclusions of the article in any way. The original article has been updated.

